# Assaying antigen-specific T cell trans-endothelial migration *in vitro* with the Transwell system: application in tumor immunology

**DOI:** 10.52601/bpr.2024.240032

**Published:** 2025-06-30

**Authors:** Ruochan Zhang, Longchao Liu

**Affiliations:** 1 CAS Key Laboratory of Pathogenic Microbiology and Immunology, Institute of Microbiology, Chinese Academy of Sciences, Beijing 100101, China

**Keywords:** Antigen-specific CD8^+^T cell, Endothelium, Migration, Chemotaxis

## Abstract

T cells play a key role in tumor immune surveillance. Trafficking of T cells to the tumor microenvironment is critical for the success of cancer immunotherapies. The process of T cell trafficking involves a sequence of steps: initial rolling along the endothelium, firm adhesion, subsequent extravasation, and directed chemotactic movement toward the tumor site. It is emerging that tumor vasculature constitutes an important barrier to T cell infiltration. In this protocol, we summarize a method for assessing antigen-specific CD8^+^T cell trans-endothelial migration *in vitro* using the Transwell system. This technique is vital for studying the mechanisms of T cell extravasation and their interactions with the vascular endothelium, and provides a controllable experimental setup to investigate T cell trans-endothelial migration.

## INTRODUCTION

Effective immune checkpoint blockade (ICB) immunotherapy depends on the intra-tumoral accumulation of T cells, especially tumor antigen-specific T cells (Liu *et al.* 2022). Tumor blood vessels are abnormal, both structurally and functionally relative to those of nonmalignant tissues, restraining the effector T cell infiltration (Johansson-Percival *et al.* 2018). Migration of T lymphocytes across the endothelium is one of the key processes in the cancer-immunity cycle, which is essential for immune surveillance and the delivery of effector T cells to tumor sites (Mellman *et al.* 2023; Vestweber 2015).

The Transwell system is a widely used tool in cell biology, and it offers a controlled *in vitro* environment to study cell migration and invasion. It consists of a microporous membrane that separates two compartments, allowing the precise manipulation of experimental conditions and the direct observation of cell behavior. By employing the Transwell system, researchers can mimic the physiological barriers that T cells encounter as they migrate from the bloodstream into tissues, including the tumor microenvironment. Moreover, researchers could also use the Transwell system and a mouse lymphatic endothelial cell line SVEC4-10 to analyze T lymphocyte migration across lymphatic endothelium (Ledgerwood and Bromberg 2007; Ledgerwood *et al.* 2008; Xiong *et al.* 2017).

In this protocol, we utilize the Transwell system to analyze antigen-specific CD8^+^T lymphocyte migration across the C166 endothelial cells. The C166 endothelial cell line, which is derived from mouse liver sinusoidal endothelial cells, exhibits normal endothelial characteristics, such as rearrangement into tubelike structures when placed on Matrigel, expression of angiotensin converting enzyme (ACE), retention of cobblestone morphology at confluence, and constitutively express vascular cell adhesion molecule-1 (VCAM-1) and the vascular addressin MECA-99 (Wang *et al.* 1996). The C166 cell line has emerged as a valuable model for studying the extravasation of immune cells in the context of tumor biology (Ahn *et al.* 2010; Huang *et al.* 2021; Nambiar *et al.* 2019; Zhang *et al.* 2021, 2023). The use of the Transwell system with C166 endothelial cells allows for the investigation of T cell behavior under various conditions, such as the presence of chemokines, cytokines, and other factors that may influence T cell extravasation and trafficking to tumor sites.

Here, we provide a detailed protocol to utilize an accessible and workable method to assay antigen-specific CD8^+^T cell trans-endothelial migration *in vitro*. By examining the roles of adhesion molecules, chemokine signals and other soluble factors, we hope to uncover the critical pathways that regulate T cell trafficking in the tumor microenvironment with this *in vitro* model. We believe that this protocol will contribute to a deeper understanding of the interplay between T cells and the endothelium during tumor progression.

## OVERVIEW OF THE PROTOCOL

C166 cells (mouse endothelial cell line) were cultured with DMEM complete medium and B16-OVA cells (mouse tumor cell line) conditioned medium (CM) for 48 hours. Next, the C166 cells were trypsinized, washed and seeded (7.5 × 10^3^ to 1 × 10^4^ live cells each) on the upper surface of a Transwell insert that had been previously coated with 0.1% gelatin. C166 cells were cultured for 16 to 24 hours to form a proper density monolayer, which was verified by staining and visual inspection using microscopes. Equal numbers of OVA_257–264_ peptide-activated OT-I CD8^+^T cells (5 × 10^5^) were added to the upper chamber of each Transwell. The lower chamber was filled with RPMI 1640 complete medium supplemented with chemoattractant CXCL10. After a 4-hour incubation, cells in the lower chamber were collected, and the number of cells that migrated was counted and analyzed.

## STEP-BY-STEP PROCEDURE

### Step 1: Cell culture [TIMING 3–5 d]

(1) To mimic the impact of the tumor microenvironment on vascular endothelial cells, this protocol utilizes B16-OVA tumor cells-derived conditioned medium to treat C166 endothelial cells (Zhang *et al.* 2023).

(2) To study the trans-endothelial activity of antigen-specific CD8^+^T cells in the tumor microenvironment, this protocol utilizes purified naїve OT-I CD8^+^T cells from OT-I mice, which are C57BL/6J background and transgenic for the T cell receptor (TCR) recognizing the ovalbumin (OVA) peptide with amino acid residues 257–264 (SIINFEKL) presented by the MHC class I molecule H-2Kb.

#### Step 1.1: Tumor cell preparation

(1) Culture the B16-OVA cells with DMEM complete medium.

(2) Collect the supernatant of B16-OVA cells in the logarithmic growth phase into 15 mL centrifuge tubes, and spin at approximately 10,000 r/min for 10 min.

(3) Collect the supernatant after centrifugation. If the cell supernatant is not used immediately, store it in −80°C.

#### Step 1.2: Endothelial cell preparation

(1) Culture the C166 cells with DMEM complete medium.

(2) Add 0.25% Trypsin-EDTA solution to the plate and observe cells under an inverted microscope until C166 cell layer is dispersed. Add adequate DMEM complete medium complete and aspirate cells by gently pipetting.

(3) Count cell number and Centrifuge cells at 1000 r/min for 5 min.

(4) Seed 1 × 10^5^ to 1.5 × 10^5^ C166 cells per well onto a 6-well cell culture plate, incubate at 37°C, 5% CO_2_, and observe the growth density.

(5) When the growth density reaches about 30%, remove and discard the culture medium, then add 2 mL of the supernatant from B16-OVA tumor cells per well and culture at 37°C, 5% CO_2_ for a 48-hour pre-treatment.

**Figure 1:**
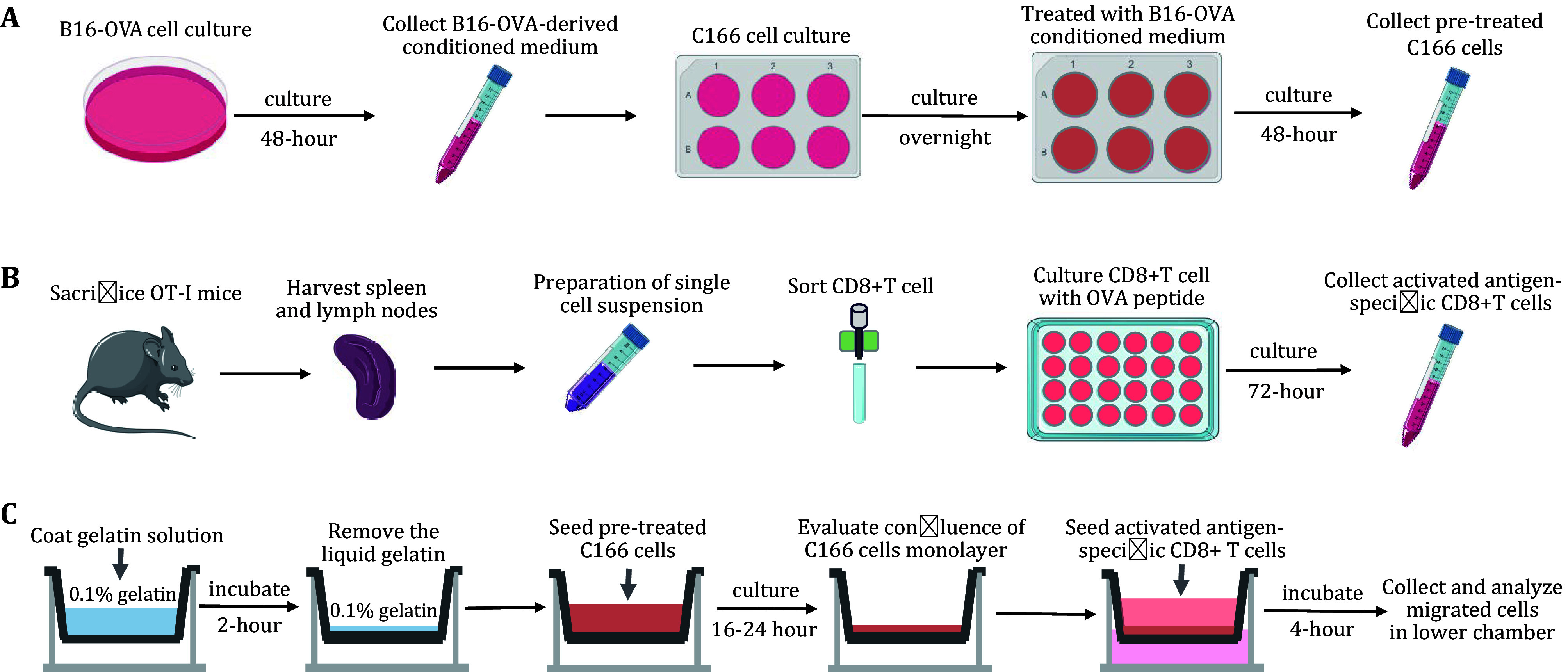
Workflow of the assay. **A** Preparation for pre-treated C166 cells. **B** Preparation for antigen-specific CD8^+^T cells. **C** The confluence of C166 monolayer and trans-endothelial migration

**[Note]** The workflow of preparation for tumor cells and pre-treated C166 cells is displayed in Fig. 1A.

#### Step 1.3: Antigen-specific CD8^+^T cell preparation

##### Step 1.3.1: Cell suspension preparation

(1) Sacrifice one OT-I transgenic mice.

(2) Carefully dissociate spleen and lymph nodes and remove stromal components using clean scissors and tweezers in sterile conditions.

(3) Place the spleen and lymph nodes into a 35 mm cell culture dish containing sterile cold 1× PBS.

(4) Rinse the spleen and lymph nodes with 2 mL of 1× PBS containing 1% penicillin-streptomycin three times.

(5) Place a 70 μm cell strainer into a 50 mL centrifuge tube and add 1 mL of RPMI 1640 medium to rinse the strainer.

(6) Place the spleen and lymph nodes to the 70 μm cell strainer, gently grind with the 1 mL syringe plunger or a pestle.

(7) Wash the strainer with 20 mL of RPMI 1640 medium and collect. Centrifuge cells at 500*g* for 5 min.

(8) Discard supernatant, treat cells with 1 mL of ACK Red Blood Cell lysing buffer and gently mix, then stand for 1 min at room temperature.

(9) Add 20 mL of RPMI 1640 medium to stop lysis, centrifuge at 500*g* for 5 min.

(10) Discard supernatant. Wash cells with 10 mL of RPMI 1640 medium and centrifuge at 500*g* for 5 min.

##### Step 1.3.2: Magnet-activated cell sorting (MACS)

(1) Count cells using 0.04% Trypan Blue (Trypan Blue is excluded by live cells).

(2) Resuspend cells with 40 μL of MACS buffer per 10^7^ cells.

(3) Add 10 μL of Biotin-Antibody Cocktail per 10^7^ cells, gently mix, and incubate at 4°C for 5 min.

(4) Add 30 μL of MACS buffer per 10^7^ cells.

(5) Add 20 μL of Anti-Biotin-Microbeads per 10^7^ cells, gently mix and incubate at 4°C for 10 min.

(6) Add 100 μL of MACS buffer per 10^7^ cells to dilute the cells.

(7) Place MS column in the magnetic field of MACS separator and place a 15 mL centrifuge tube below for collecting cells.

(8) Add 1 mL MACS buffer to rinse the column, and then apply cell suspension onto the column. Collect the flow-through cells (representing the purified CD8a positive cells).

(9) Wash the column with 500 μL of MACS buffer three times and collect all the flow-through unlabeled cells.

(10) Count the collected flow-through cells below.

##### Step 1.3.3: Antigen-specific CD8^+^T cell activation

(1) Resuspend purified OT-Ⅰ CD8^+^T cells with RPMI 1640 complete medium at a concentration of 5 × 10^5^ cells/mL, with supplying 200 ng/mL OVA_257–264_ peptide.

(2) Seed 1 mL of OT-I CD8^+^T cells suspension (5 × 10^5^ cells) per well onto a 24-well cell culture plate.

(3) Incubate at 37°C, 5% CO_2_ for three days and keep track of the cell expansion.

**[Note]** The workflow of preparation for antigen-specific CD8^+^T cells is displayed in Fig. 1B.

#### Step 1.3 (Optional): Alternative approach for Antigen-specific CD8^+^T cell preparation

We also provide an alternative approach to acquiring abundant antigen-specific CD8^+^T cells, which does not require to isolate CD8^+^T cells. The proportion of CD8^+^T cells in spleen and lymph node of OT-I TCR transgenic mouse is approximately 10% to 20% and they will expand *in vitro* during the following steps.

(1) Prepare a suspension of the spleen and lymph nodes according to Step 1.3.1.

(2) Resuspend OT-I cells with RPMI 1640 complete medium at concentration of 1 × 10^6^ cells/mL, with supplying 200 ng/mL OVA_257–264_ peptide.

(3) Seed 1 mL of OT-I cells suspension (1 × 10^6^ cells) per well onto a 24-well cell culture plate. Incubate at 37°C, 5% CO_2_. Passage cells every two days by splitting 500 μL of cell suspension from each well and placing them in new wells, then filling with 500 μL of fresh RPMI 1640 complete medium.

(4) After 5 to 7 days, the harvested cells should predominantly consist of over 80% to 90% CD8^+^T cells.

**[Note]** Examining the proliferation and cell death of antigen-specific CD8^+^T cells is important. We recommend optimize the culture condition of antigen-specific CD8^+^T cell by measuring cell proliferation (such as CFSE staining) and cell death (such as Annexin/PI staining). Change culture medium frequently helps to maintain the metabolic activity of T cells. Furthermore, supplement with the cytokine IL-7 and IL-15 is also an option to sustain the survival and proliferation of CD8^+^T cells.

### Step 2: Prepare confluence of endothelial cells [TIMING 2 d]

(1) Check the viability and confluence of the pre-treated C166 endothelial cells in different culture mediums.

(2) Add 100 μL of 0.1% gelatin solution into each Transwell inserts chambers of a 24-well cell culture plate, incubate at 37°C, 5% CO_2_ for 2 h.

(3) Collect and count the pre-treated endothelial as previously mentioned. Resuspend cells at a concentration of 7.5 × 10^4^ to 1 × 10^5^ cells/mL.

(4) Remove Transwell plate from incubator. Use a pipette to carefully remove the liquid gelatin overlying inserts, being cautious not to scratch or puncture the membrane.

(5) Add 100 μL of the resuspended cells (7.5 × 10^3^ to 1 × 10^4^ cells per well) into the 0.1% gelatin-coated Transwell inserts.

**[Note]** We advise doing triplicates for each condition.

(6) Incubate at 37°C, 5% CO_2_ for 16 to 24 hours until the C166 cells form a dense endothelial cell monolayer.

### Step 3: Evaluate the confluence of C166 cells monolayer on Transwell inserts [TIMING 1 h]

A confluent monolayer is crucial for the experiment. This protocol advises seeding 7.5 × 10^3^ to 1 × 10^4^ cells per well into the 0.1% gelatin-coated Transwell inserts for 16 to 24 hours until the C166 cells form a confluent monolayer. It is essential to perform a gradient dilution pre-test, which determines the optimal cell seeding density and incubating time. Overgrowth of C166 cells can impede T-cell migration through the monolayer. Conversely, large gaps within the C166 cell monolayer may lead to excessive migration of T cells, artificially elevating the measured numbers of the transmigrated cells.

**Figure 2:**
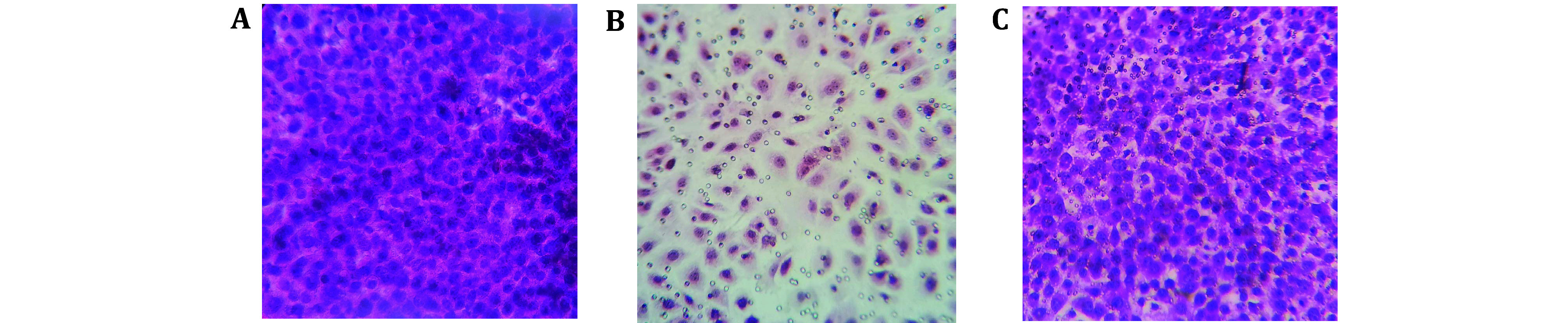
Assessment of C166 monolayer confluence on the Transwell inserts. H&E staining of C166 endothelial cell layers on the Transwell inserts coated with 0.1% gelatin. Panel A depicts a monolayer that is overly dense, Panel B shows a layer that is too sparse, and Panel C presents an optimal confluence that is considered appropriate for the assay

(1) Remove the Transwell plate from the incubator. Remove a Transwell insert from the 24-well cell culture plate.

(2) Use a pipette to carefully remove the cell medium within the insert.

(3) Add 100 μL of methanol into the Transwell insert to fix for 30 s.

(4) Add 100 μL of Hematoxylin into the Transwell insert to stain for 30 s and rinse with water.

(5) Carefully remove the mesh membrane from the Transwell insert using a sharp blade without damaging the cell monolayer.

(6) Place mesh membrane on a slide, add a drop of neutral resin and cover with a coverslip.

(7) Observe and assess the confluence of the C166 endothelial cell layer under a light microscope. If the cells have reached confluent, proceed to the trans-endothelial migration assay. The representative images of the stained C166 endothelial cell layer are displayed in Fig. 2.

### Step 4: Migration assay [TIMING 6 h]

(1) After evaluating the suitable confluence of the C166 monolayer as above, remove the Transwell insert from the 24-well cell culture plate, exposing the wells from the receiving plate.

(2) Add 600 μL of RPMI 1640 complete medium with appropriate concentration of chemokine to the bottom of the well.

**[Note]** This protocol uses 250 ng/mL recombination mouse CXCL10 for chemotactic signal (Nambiar *et al.* 2019). Other chemokines can also be applied according to the purpose of different experiment.

(3) Carefully place the Transwell insert onto the receiving plate. The liquid from each well will be in contact with the Transwell insert. Check for bubbles in the contact surface.

**[Note]** The presence of bubbles on the contact surface can affect chemotaxis, hence it is necessary to check for and address them.

(4) Resuspend with RPMI 1640 complete medium at a concentration of 5 × 10^6^ cells/mL.

(5) Add 100 μL of prepared antigen-specific CD8^+^T cells suspension (5 × 10^5^ cells) to each upper chamber of the Transwell insert.

(6) Close the plate and place it in the incubator at 37°C, 5% CO_2_ for 4 h.

(7) After the incubation time, carefully remove the Transwell insert away from the receiving plate.

(8) Collect the cells from the lower chamber of the receiving plate.

(9) Count and analyze the number and phenotype of the transmigrated CD8^+^T cells.

**[Note]** (1) Evaluating the cell death of the transmigrated CD8^+^T cells is necessary. Count cells using 0.04% Trypan Blue or exclude dead cells from flow cytometry analysis using viability dyes. (2) The percentage of T-cell migration can also be calculated according to the requirements. (3) The workflow of preparation for the confluence of C166 cell monolayer and trans-endothelial migration antigen-specific CD8^+^T cells is displayed in Fig. 1C.

## TROUBLESHOOTING

Troubleshooting advices can be found in Table 1.

**Table 1:** Troubleshooting in this protocol

Step	Problem	Possible reason and solutions
Step 1	C166 cells grow slowly and are in poor condition.	Excessive passaging and cellular senescence may lead to poor condition of cells. Ensure that cells have not undergone excessive passages.
Step 2	C166 cells do not reach confluence on Transwell insert.	The number of C166 cells added or culture period is not enough. Ensure that sufficient numbers of C166 cells were seeded onto the Transwell.
Step 4	Very small number of T cells undergo transmigration to lower compartment.	(1) Ensure that cell counts are correct. (2) Ensure that appropriate confluence of C166 cells monolayer. (3) Ensure chemokine concentrations are sufficient to induce chemotaxis of T cells.

## ANTICIPATED RESULTS

**Figure 3:**
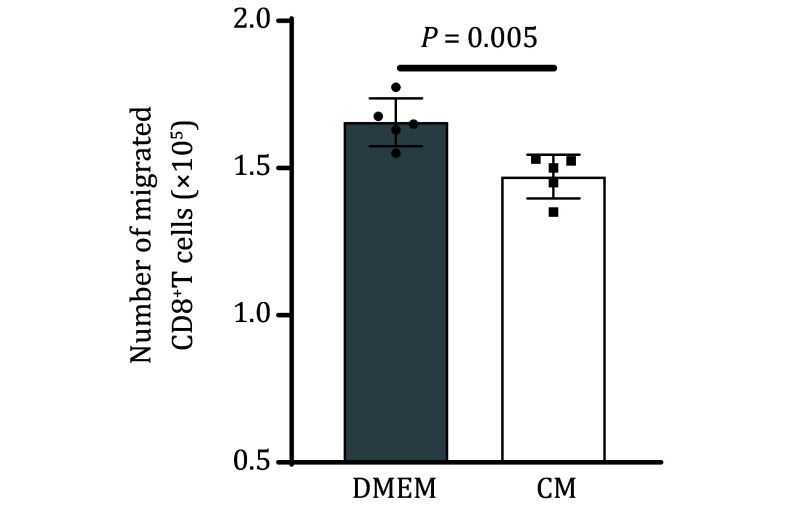
Analysis of migrated CD8^+^T cells in the lower chamber. The bar graph shows the transmigrated numbers of total antigen-specific CD8^+^T cells in the context that C166 endothelial cells pre-treated with B16-OVA conditioned medium (CM) or DMEM complete medium

(1) Following the protocol, we stained and observed the confluence of the C166 endothelial cell layer under a light microscope. If the cells have reached evenly confluent, proceed to the trans-endothelial migration assay. Figure 2 shows representative images of the stained C166 endothelial cell layer.

(2) Following the protocol, 15% to 30% of antigen-specific CD8^+^T cells placed into the upper chamber of the Transwell insert will migrate into the lower chamber. The bar graph of Fig. 3 shows the migrated numbers of total antigen-specific CD8^+^T cells with indicated treatment.

## ADVANTAGES OF THIS PROTOCOL

This protocol allows researchers to manipulate specific variables and study their effects on immune cell migration under defined conditions. Researchers can customize this protocol to suit specific research questions by altering the endothelial cell type, immune cell subset, chemoattractants, and other experimental conditions. These assays help in dissecting the molecular and cellular mechanisms involved in Trans-endothelial migration, including the role of adhesion molecules, chemokines, and signaling pathways. Compared with other *in vitro* chemotaxis assays for a single component of tumor cell lines or immune cells, this protocol provides an experimental paradigm that more closely mimics the physiological conditions of the tumor microenvironment, which T cells migrate across and interact with the vascular endothelium to respond to the chemotactic mediators. Furthermore, this protocol is reproducible, relatively simple to set up and execute, and provides results in a shorter time frame compared to *in vivo* models.

## LIMITATIONS OF THIS PROTOCOL

This protocol is performed under static conditions without flow, which may not accurately represent the tumor environment *in vivo* where blood flow can influence cell behavior and interactions. The *in vitro* models may not fully capture the complexity of the *in vivo* microenvironment, including the presence of extracellular matrix components, multiple cell types, and other soluble factors. The use of endothelial cell lines may not fully mimic the biological properties of native endothelial surfaces, especially tumor endothelium. Researchers must consider these limitations when planning experiments and interpreting the results, and they may need to combine *in vitro* assays with *in vivo* studies to comprehensively understand the mechanisms of immune cells trans-endothelial migration in tumors.

## MATERIALS

Materials used in this protocol are listed in Table 2 and Table 3.

**Table 2:** Key resources used in this protocol

Reagent or resource	Source	Identifier
Cell lines		
C166	American Type Culture Collection (ATCC)	CRL-2181
B16-OVA	National Infrastructure of Cell Line Resource (NICR)	
Reagents		
Dubelcco’s Modified Eagle’s Medium (DMEM)	Gibco	Cat. #C11995500BT
RPMI 1640 medium	Gibco	Cat. #C11875500BT
Penicillin-Streptomycin (P/S, 10000 U/mL)	Gibco	Cat. #15140-122
Fetal bovine serum (FBS)	HyClone	Cat. #SH30087.03
β-Mercaptoethanol (1000×)	Gibco	Cat. #21985023
Non-Essential Amino Acids Solution (NEAA, 100×)	Gibco	Cat. #11140050
Sodium pyruvate (100×)	Gibco	Cat. #11360070
HEPES	Gibco	Cat. #15630-080
DMSO	Solarbio	Cat. #D8371
OVA_257-264_ peptide (SIINFEKL)	Chinese peptide	Custom synthesis
0.25% Trypsin–EDTA	Gibco	Cat. #25200056
Trypan Blue Stain	Gibco	Cat. #15250061
1× PBS	YEASEN	Cat. #41403ES76
0.1% (*wt*/*vol*) gelatin	Precision BioMedicals	Cat. #211050
Red Blood Cell lysing buffer	Solarbio	Cat. #R1010
Mouse CD8a^+^T Cell Isolation Kit	Miltenyi Biotec	Cat. #130-104-075
Mouse recombinant CXCL10	Peprotech	Cat. #250-16
0.5 mol/L EDTA (PH8.0)	Solarbio	Cat. #E1170
BSA	Beyotime	Cat. #ST023
Methyl alcohol	Macklin	Cat. #M813895
Hematoxylin and Eosin Stain kit	Solarbio	Cat. #G1120
Equipment and Consumable		
Benchtop centrifuge	cence	L550
Light microscope	Leica	
Biological safety cabinet	Haier	HR40-IIA2
Carbon dioxide incubator	SANYO	MCO-15AC
Water bath	MIULAB	WT100-1B
Automated cell counter	Nexcelom Bioscience	Cellometer Mini
Cell counting chamber	Nexcelom Bioscience	Cat. # SD100
Pipettes: 10 μL, 200 μL,1 mL	Eppendorf	Cat. # T090RS
0.22 μm filter	SAINING	Cat. #5032000
15 mL centrifuge tube	FDCELL	Cat. # L030015
50 mL centrifuge tube	FDCELL	Cat. # L030050
100 mm cell and tissue culture plates	Corning	Cat. # 430167
35 mm cell and tissue culture plates	Corning	Cat. # 430165
6-well cell culture plates	Corning	Cat. # 3516
24-well cell culture plates	Corning	Cat. # 3524
70 μm cell strainer	BD Falcon	Cat. # 352350
1 mL sterile syringe	BD Falcon	Cat. # 300841
Sterile cell mesh pestle	JETBIOFIL	CSP001001
6.5 mm Transwell with 5.0 μm pore polycarbonate membrane insert	Corning	Cat. # 3421
MACS MultiStand	Miltenyi Biotec	Cat. #130-042-303
MiniMACS Separator	Miltenyi Biotec	Cat. #130-042-102
MS column	Miltenyi Biotec	Cat. #130-042-201

**Table 3:** The medium and buffer solutions used in this protocol

Solution	Formulation
DMEM complete medium	Add 10% FBS and 1% P/S to DMEM medium. This solution can be stored at 4°C.
RPMI 1640 complete medium	Add 10% FBS, 1%P/S, 0.1 mmol/L NEAA, 1 mmol/L sodium pyruvate, 10 mmol/L HEPES, 50 μmol/L β-mercaptoethanol to RPMI 1640 medium. This solution can be stored at 4°C.
10% BSA stock	Dissolve 5 g BSA in 50 mL deionized water and filter with the 0.22 μm filter. This solution can be stored at −20°C.
MACS buffer	Add 1% BSA and 2mM EDTA to 1×PBS. This solution can be stored at 4°C.

## Conflict of interest

Ruochan Zhang and Longchao Liu declare that they have no conflict of interest.
